# The acceptability, adoption, and feasibility of a music application developed using participatory design for home-dwelling persons with dementia and their caregivers. The “Alight” app in the LIVE@Home.Path trial

**DOI:** 10.3389/fpsyt.2022.949393

**Published:** 2022-08-18

**Authors:** Line Iden Berge, Marie Hidle Gedde, Juan Carlos Torrado Vidal, Bettina Husebo, Kia Minna Hynninen, Solgunn Elisabeth Knardal, Kristine Gustavsen Madsø

**Affiliations:** ^1^Norske Kvinners Sanitetsforening (NKS) Olaviken Gerontopsychiatric Hospital, Askøy, Norway; ^2^Department of Global Public Health and Primary Care, Faculty of Medicine, Center for Elderly and Nursing Home Medicine, University of Bergen, Bergen, Norway; ^3^Haraldsplass Deaconess Hospital, Bergen, Norway; ^4^Municipality of Bergen, Bergen, Norway; ^5^Department of Clinical Psychology, Faculty of Psychology, University of Bergen, Bergen, Norway

**Keywords:** dementia, home-dwelling, music interventions, application, participatory design, acceptability, adoption

## Abstract

**Background:**

Music interventions for persons with dementia can improve health and interaction with caregivers, yet the reach is often restricted to institutions. We describe the participatory design process of a prototype music application for patients affiliated with a gerontopsychiatric hospital and evaluate the acceptability, adoption, and feasibility of use for dyads of home-dwelling persons with dementia and their informal caregivers.

**Methods:**

The application “Alight” was developed following an iterative, expert-driven participatory design approach, which includes a requirement elicitation phase and two rounds of prototyping and testing in real-world settings. End users and stakeholders were involved in all steps, that is, workshops, interviews, field observation, ethnographic inquiries, and beta testing sessions with music therapists, patients, and caregivers in collaboration with a commercial music and technology company. The last prototyping and testing took place in the LIVE@Home.Path trial, a stepped-wedge multicomponent randomized controlled trial to improve resource utilization and caregiver burden in municipal dementia care during 2019–2021.

**Results:**

Mean age of the person with dementia in the LIVE@Home.Path trial was 82 years, 62% were female, and the majority had Alzheimer's dementia (44%) of mild severity (71%). Sixty-three dyads were offered Alight in the multicomponent intervention, of which 13% (*n* = 8) accepted use. The dyads accepting Alight did not differ in demographic and clinical characteristics compared to those not interested. The feasibility was high among those accepting Alight, 75% (*n* = 6) reported a positive impact on mood, 50% (*n* = 4) experienced a positive impact on activity, and 50% (*n* = 4) gooduser-friendliness. The adoption was high with daily use or use several times a week reported by 63% (*n* = 5). Obstacles emerged when updating the application in homes without wireless Wi-Fi, and some participants were unfamiliar with using touchscreens.

**Conclusion:**

The feasibility and adoption of the application were high and accepting dyads did not differ on demographic and clinical variables from those not reached. This suggests a high potential for utilization in dementia care. This study contributes methodologically to the field of participatory design and mHealth interventions by demonstrating a specific design approach that throughout the process successfully involved researchers, industry partners, health care practitioners, and end users.

**Clinical trial registration:**

ClinicalTrials.gov, NCT04043364.

## Introduction

Dementia is a progressive syndrome characterized by cognitive impairment inferring with daily living, and changes in personality, mood, and behavior (1). As the world population is aging, the prevalence of dementia increases, and today, about 55 million people are affected, making it the leading cause of disability and loss of independence in old age (1). Dementia is the only top ten cause of death globally that cannot be significantly prevented, cured, or modified (2), leaving disease management, caregiver support, and service innovation as the main targets for the reduction of the disease burden (3). Music intervention is a promising rehabilitation strategy not only for dementia, but for several neurological disorders, including strokes, Parkinson's disease, epilepsy, and multiple sclerosis (4). Musical memory of familiar songs and genres are often well-preserved over the dementia course and may catch attention and improve mood, while the personal connections with music may also support a positive sense of identity even in later stages of the disease (5). This is reflected by findings from a recent Cochrane review, which concludes that music-based therapeutic interventions reduce depressive and overall behavioral symptoms in persons with dementia (PwD), while also improving their wellbeing and quality of life (6). Likewise, evidence from randomized controlled trials suggests that music-based interventions can support caregivers of PwD. Regular musical activities with singing and listening to familiar songs enhance the wellbeing of caregivers to PwD receiving daycare, while music therapy programs in care homes support caregiving techniques and improve communication with staff (7). A recent review highlights the need for widely available and easily implemented music interventions for people with neurological disorders, such as dementia, and suggests that mobile applications, should play a key role in providing music in hospitals, community institutions, and to patients residing at home (4).

eHealth is a broad term referring to the cost-effective and secure use of information and communication technologies to support health, including health care services, surveillance, education, and research (8). A subset of eHealth, mobile Health (mHealth), is defined by the World Health Organization as “Medical and public health practice supported by mobile devices, such as mobile phones, patient monitoring devices, personal digital assistants, and other wireless devices” (9). mHealth may increase the reach of music interventions to PwD, and provide an excellent opportunity to safeguard living at home with dementia through increased reach and cost-effectiveness of services combined with more individualized and precision-based care. One example is to promote health through applications for smartphones and tablets, and today, several hundreds of applications concerned with dementia are commercially available in Apple App Store and Google Play Store (10).

Applications targeting PwD and caregivers can efficiently provide remote health services encompassing screening and training of cognitive function, monitoring safety and navigation, as well as enabling socialization with family and friends (11). Applications for caregivers can additionally offer mental support and platforms for communication with health care professionals and peers (12). A systematic review concluded that mHealth interventions could improve health for persons with mild cognitive impairments and dementia, yet, the majority of the studies were of low quality (13). More recently, one mixed-method cohort study has been added to the literature examining the use of a music application to promote song-task associations in PwD residing in care homes (14). Even though no change in quantitative measures of wellbeing and quality of life were detected after several weeks of use, the interview of staff suggested positive changes in behavior and routine in the residents daily living (14). We are not aware of any comparable studies on music applications designed for home-dwelling PwD, despite that this group constitutes an increasing market for developers. Nonetheless, sustainable implementation of mHealth applications at home for PwD is demanding. A systematic review of qualitative studies concluded that even though this technology could enhance health by stimulating cognitive function and communication, implementation was challenged by PwD's digital literacy and the design of the application, highlighting the need for involving users early in the design process (15).

As such, mitigating barriers for the implementation of applications by the user-centered development can increase the reach of music interventions regardless of care level. Participatory, or co-design, describes an increasingly popular approach for designing mHealth systems, which involve the end users and their stakeholders to a greater extent, preferably in all stages of the iterative development process (16, 17). Whereas some participatory design approaches in mHealth are most dependent on the expertise of engineers, others rely most profoundly on clinical professionals, i.e., the domain expert. Systematic reviews suggest that participatory design provides an opportunity to develop acceptable and feasible mHealth interventions for vulnerable groups in general (18) and more specifically, by enhancing de-stigmatization and empowerment for PwD (19).

This study (1) describes the development of a prototype of a music application for patients in a gerontopsychiatric hospital following an expert-driven participatory design process and (2) evaluates the acceptability, adoption, and feasibility of use for dyads of home-dwelling persons with dementia and their informal caregivers in a clinical trial in municipal dementia care.

## Methods

### Development of the Alight application for tablets

**Figure 1:**
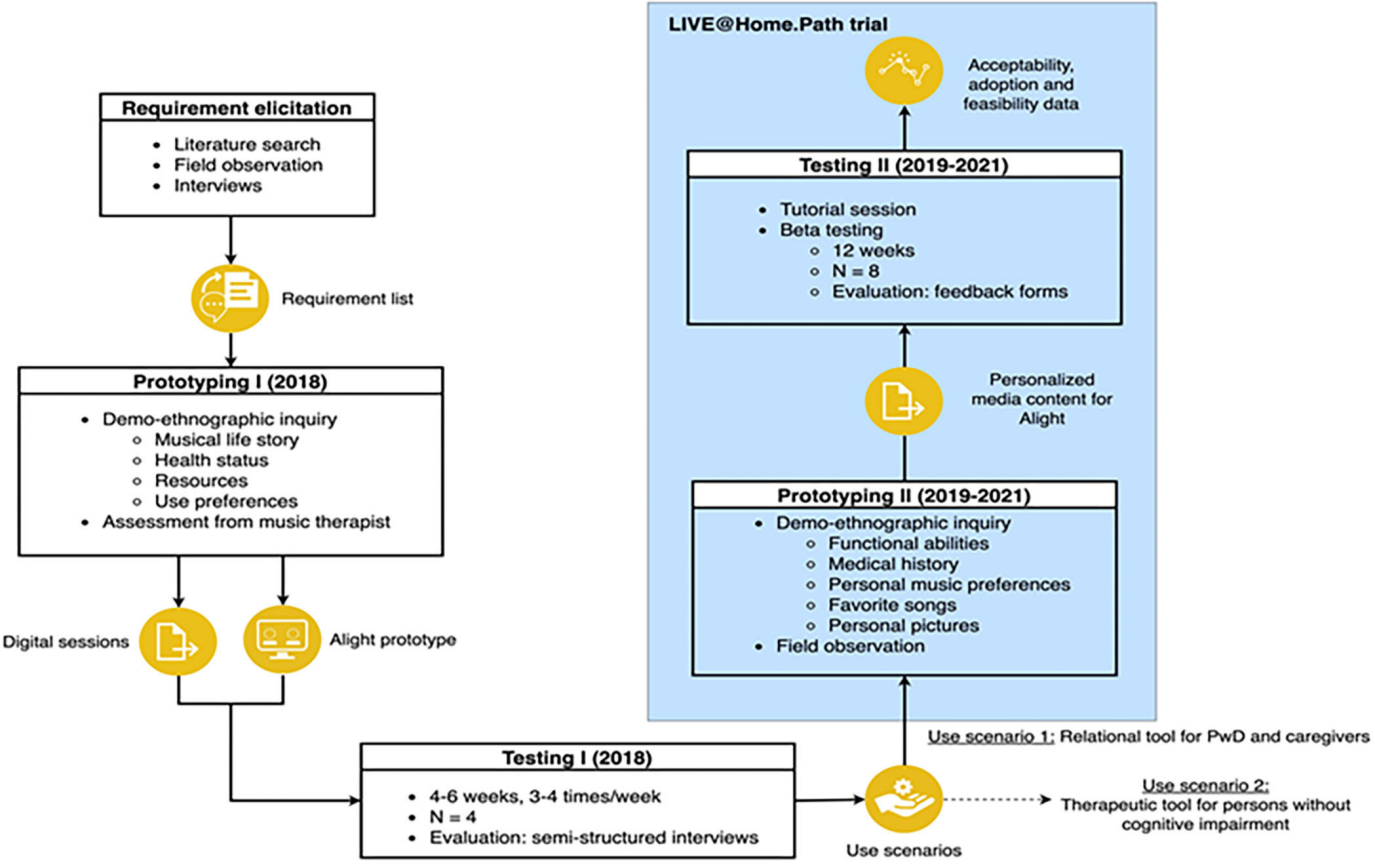
The iterative participatory design process of the Alight application.

This section describes the participatory design approach to create Alight, an mHealth application for iOS systems that delivers personalized music interventions to older adults (Figure 1). This participatory design process is grounded in Demerbileks model for product design for elderly people encompassing Usability, Safety, and Attractiveness Participatory design (USAP), and suggests two phases generating both a conceptual and a refined prototype (20).

NKS Olaviken Gerontopsychiatric Hospital offers specialist health care services in old age psychiatry and dementia care for the region of Bergen, Norway. It comprises three wards with a total of 21 beds, the average length of stay is 6 weeks, and serves ~160 inpatients per year, which equals 6,500 bed days. The hospital provides patient-centered treatment through multiprofessional teams, including physicians, psychologists, nurses, occupational, and music therapists. In this context, the concept of the Alight application originated. In 2016, contact was established with Soundio (21), a creative music and technology company *via* a regional innovation facilitation company, VIS (22). A requirement elicitation phase started in 2017, in which team members from Soundio observed sessions with music therapist and patients, and interviewed users, caregivers, and staff about their needs and preferences for applications supporting music interventions for older adults. Additionally, we carried out two workshops with all the above-mentioned participants, structured as a focus group, in which decisions regarding user scenarios and design were discussed.

The list of requirements and needs emerging from this process was used in the first prototyping stage in 2018 (Figure 1). Based on the music therapist's assessment including the patients “musical life story”, health status, resources, and preferences for use, Soundio designed digital sessions of 15–20 min duration in the prototype application Alight. The digital sessions were created using iMovie combined with the music bought from iTunes, personal pictures, and other non-licensed pictures on the internet to illustrate time periods and places of significance to the users. The sessions also allowed for the inclusion of personalized components, such as familiar pictures and videos, relaxation exercises, and vocalization training. Next, we tested the use of the application in two men and two women aged 55 years and above with psychiatric and neurological disorders, including dementia. They utilized the sessions 2–3 times a week for 4–6 weeks, either at home or in nursing homes, using it individually or together with formal or informal caregivers. After the test period, the music therapist performed interviews with patients and formal/informal caregivers applying a self-developed interview guide (Supplementary material 1). A woman hospitalized for a major depressive episode reported that the application enabled her to continue the inpatient music therapy at home. A man with dementia permanently residing in a nursing home experienced that the application facilitated conversations with staff with different and more meaningful content than usual, while the staff reported that the application allowed for a new type of acquaintance with him. A woman with an affective disorder and a man with a neurological disorder with cognitive and functional impairment corresponding to dementia syndrome both utilized the application in the transition phase from hospital to nursing home. They reported that they enjoyed the content of the application, but experienced difficulties managing it independently due to their functional disabilities, which minimized use in a buzzy ward. These experiences guided further development and the determination of two distinct use scenarios of the application: (1) therapeutic tool for older adults with minimal cognitive impairment to support the continuation of inpatient music therapy after discharge; (2) relational tool, a “digital music memory book” for persons with dementia, supporting positive interactions with formal and informal caregivers.

### The LIVE@Home.Path trial

During 2019–2021, the use of Alight was explored as a “digital music memory book” in the LIVE@Home.Path trial, a stepped wedge randomized controlled trial evaluating whether a multicomponent intervention improved resource utilization and caregiver burden in municipal dementia care (3). A stepped wedge randomized controlled trial is a one-way cross-over the trial in which all participants receive the intervention, and the timing of the intervention is determined by randomization. This stepped-wedge trial used a closed cohort design implying that all participants were recruited before randomization, exposed to both the control and the 6-month intervention period, and assessed repeatedly every 6 months. This yielded in total five cross-sectional data collections conducted in the dyad's homes at discrete time points during the 24-month trial. Dyads were eligible for inclusion if the person with dementia were ≥65 years, diagnosed with dementia with a Mini-Mental State Examination (MMSE) score of 15–26 or Functional Assessment Staging (FAST) score of 3–7, home-dwelling in one of three specified Norwegian municipalities, while inclusion criteria for the caregiver were minimum weekly face-to-face contact with the PwD. To overcome the logistical challenges of the requirement of participants from both primary and secondary health care services, we used convenience sampling to recruit dyads from geriatric or gerontopsychiatric outpatient clinics, municipal memory teams, and general media without financial incentives. The multicomponent intervention was delivered to the dyads by a municipal coordinator, which was a person with a minimum bachelor's degree in nursing, learning disability nursing, and occupational therapy already working in municipal dementia care. LIVE is an acronym for the single elements in intervention consisting of L: learning (offering learning programs for caregivers and PwD), I: innovation and information and communication technology, ICT (tailored assistive technology and telecare), V: volunteering (offering a visiting friend), and E: empowerment (medication reviews and advanced care planning at the general practitioner). While the coordinators implemented the components, participants in Bergen municipality were additionally offered to participate in further development of the “Alight” application.

### Implementation of the Alight application in the LIVE@Home.Path trial

**Figure 2:**
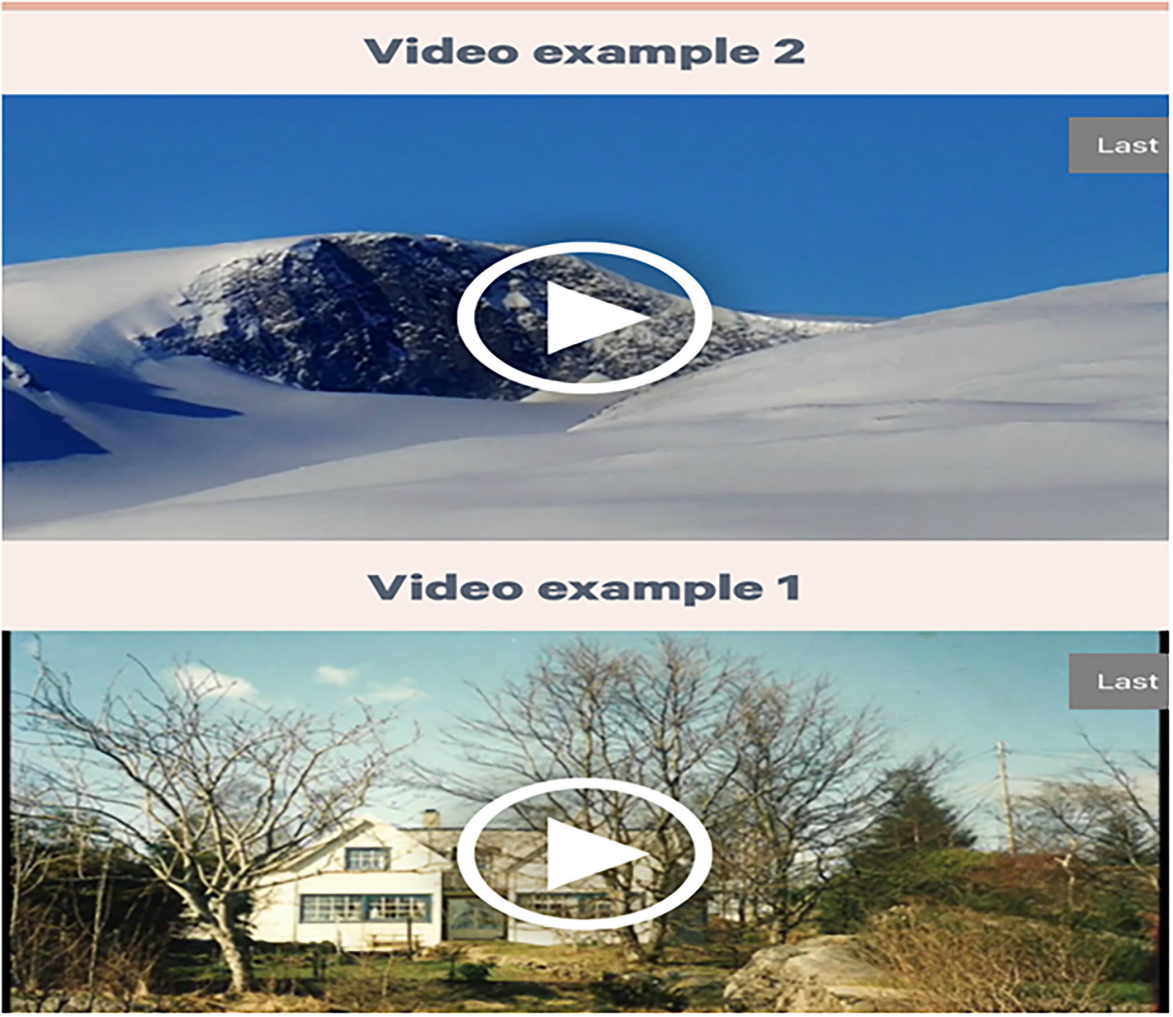
Screenshot of a launch page in the Alight application. The user presses the ≪play≫ icon to start the video.

If the dyads were interested in testing the Alight application, the coordinator provided them with written material, including contact information, to the music therapist at the NKS Olaviken Hospital, who had been crucial in the development of the prototype from 2017 to 2018. This matching between music therapists and dyads was used to carry out the second prototyping stage (Figure 1). When contact was established, the music therapist visited the dyads at home and explored the PwD's functional abilities and personal musical preferences. During visits of ~60 min duration, she invited herself to become familiar with the dyads by listening to significant stories of their life, exploring pictures of places and persons of importance, and identifying and playing favorite music together. This information was used to create the content of the application, building on knowledge concerning the therapeutic factors of music in dementia (23). After this systematic assessment, information on preferred songs and scanned copies of personal pictures was provided to Soundio pr email without other personal data. Team members from Soundio subsequently designed the video sessions in Alight, which lasted 15–30 min and utilized 4–8 songs. A screenshot of the launch page of the application is provided in Figure 2. The zipped video file was sent from Soundio to the music therapist using a Dropbox link with a password provided in a separate mail. She downloaded the video on iPads with the Alight application, while the original video was saved in a secure could-based storage system. The application was installed on the dyad's private iPad, if they had one. If not, the application was installed on an iPad owned by the hospital, which the dyads borrowed during the study. At the second home visit, the music therapist empowered the dyads to become familiar with the application and tablet, aiding them to use it offline, or online, if they had domestic WiFi.

Then, we carried out the beta testing process of the application, in which the dyads could use Alight on their own, at home, without supervision from music therapists or researchers. The application was available to the dyads for 12 weeks, and thereafter, Soundio deleted the account and video session in Alight, including the private photos. After 90 days, all personal data were deleted from the Soundios server.

### Data material and analyses

**Figure 3:**
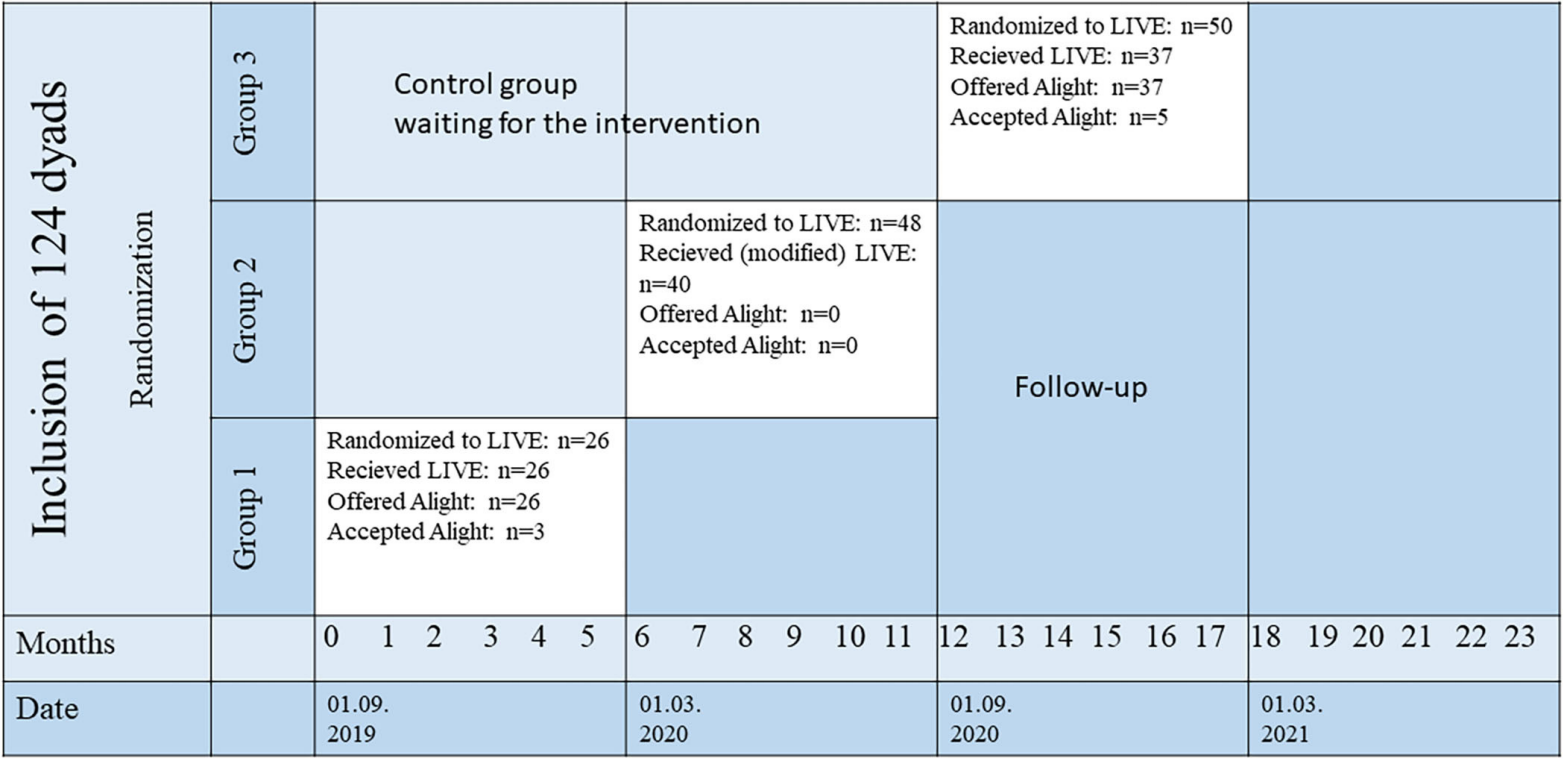
Acceptability of the Alight application in the stepped wedge randomized controlled LIVE@Home.Path trial. A total of 124 dyads of persons with dementia and their caregiver were included in Bergen municipality before 01/09/2019 and randomized in time to receive the 6-month LIVE intervention in three groups. Group 1 received the intervention from 01/09/2019 to 28/02/2020, Group 2 received a modified version of the intervention without Alight from 01/03/2020 to 31/08/2020 (COVID-19 restrictions), and Group 3 received the intervention from 01/09/2020 to 28/02/2021. Demographic and clinical data from the dyads utilized in this study were collected before start of intervention groups 1 and 3 (months 0 and 12).

This study utilized information from two sources. First, we used data from the LIVE@Home.Path to explore the acceptability of the application, estimating the rate of acceptance (number of dyads using Alight/number of dyads offered Alight) in the overall sample and stratified by cohabiting status. Furthermore, we explored if and how the characteristics of dyads using Alight differed from those who did not use Alight. We used PwDs and their caregiver's demographic and clinical variables from the data collection most proximate to the intervention period assigned to the dyads (Figure 3), and these variables were selected based on our experiences as clinicians and researchers in dementia care. The degree of cognitive impairment was evaluated with Mini-Mental State Examination (MMSE, range 0–30, a lower score indicates greater cognitive impairment) (24), and dementia severity was evaluated with the Functional Assessment Staging Test (FAST, range 1–7, a higher score indicates poorer functioning) (25), while dependency of daily living was assessed by Physical Self-Maintenance Scale (PSMS, range 6–30, higher score indicates higher dependency) and Instrumental Activities of Daily Living Scale (IADL, range 8–31, higher score indicates higher dependency) (26, 27). Neuropsychiatric symptoms were proxy rated by the caregiver using the Neuropsychiatric Inventory (NPI-12, range 0–144, a high score indicates high symptom load) (28). Participants were asked if they used assistive technology (passive sensor technologies, active sensors, and video communication). Both the patient and caregiver reported dementia-specific quality of life with Quality of Life on the Alzheimer's disease scale (29) (QoL-AD, range 13–52, high score indicates high quality of life) and generic quality of life with EQ-5D-VAS scale (range 0–100, high scores indicates high quality of life) (30). Finally, caregiver burden was assessed with the Relative Stress Scale (RSS, range 0–60, high score indicates high burden) (31), and caregiver depressive symptoms with the Geriatric Depression Scale (GDS, range 0–60, high score indicates a high symptom load) (32). We used Stata/IC, release 17 (Stata Corp LP, College Station, TX) to analyze the data with descriptive statistics, a *t*-test was used to compare the mean of continuous variables, and a chi-square test to compare categorical variables, level of significance was 0.05. Sum scores of QoL-AD, NPI, RSS, and GDS were generated if ≥80% of items were answered, otherwise, the sum score was regarded as missing.

Second, we used data from the self-developed feedback form to explore the adoption (i.e., frequency of use) and feasibility (i.e., user-friendliness, usefulness, and impact) of the application. Within 4 weeks after the 12-week test period this form was sent by postal mail to the accepting dyads, also including an envelope with the return address and stamp. As the System Usability Scale (SUS) is not adapted and validated for PwD (33), we developed a custom form based on our expert clinical experience with the patient group (Supplementary material 2). This form included questions with a fixed set of possible answers, but also enquired for general feedback on open-ended questions. Furthermore, we added the experiences from the music therapist regarding user-friendliness and obstacles to evaluate the feasibility.

### Ethics

The LIVE@Home.Path trial is approved by the Regional Committee for Medical and Health Research Ethics, North Norway (2019/385) and registered at ClinicalTrials.gov (NCT04043364). As required by the General Data Protection Regulation (GDPR), we developed a Data Protection Impact Assessment (DPIA, ePhorte UiB: 2019/5569). After providing verbal and written information, spoken and written informed consent for participation was obtained in direct conversation with the caregiver and PwD, if s/he was able to provide consent for participation. If not, the next of kin or a legal advocate provided consent based on their determination of whether the PwD would have agreed to participate, when able to consent. Soundio AS had no role in developing and conducting this study, including management of data or interpretation of the results.

## Results

A total of 280 dyads were included from the three municipalities in the LIVE@Home.Path trial, of which 124 resided in the municipality of Bergen (Figure 3). Due to the COVID-19 pandemic from March 2020, Group 2 received a postponed and slightly modified LIVE intervention (34). Dyads in this group were not offered Alight, as home visits were not feasible during the strictest physical distancing restrictions. Figure 3 shows that three of 26 dyads offered Alight accepted use in Group 1, while five out of 37 dyads in Group 3 accepted use. This yielded an overall acceptance rate of 13% (8 accepting dyads among 63 dyads offered Alight). Stratifying acceptance rate by cohabitation status gave an acceptance rate of 8.6% among dyads with PwD living alone (3 dyads accepting/35 dyads offered), while the corresponding number among the dyads with co-residing PwD and caregiver was 17.9% (5 dyads accepting/28 dyads offered), yet these two prevalence estimates were not significantly different when tested in a logistic regression model (OR = 2.32, 95% CI: 0.51–10.70, *p* = 0.281).

In the sample of dyads offered Alight (*n* = 63), the mean age of the PwD was 82.2 years, 62% were female (*n* = 39) and 56% lived alone (*n* = 35) (Table 1). The majority had mixed dementia (49%, *n* = 31), mean MMSE was 19 points, and 71% (*n* = 39) were classified with mild functional impairment equal to 3–4 points according to FAST. Participants reported a mean QoL-AD of 37.2 and an EQ-5-D VAS scale for health of 76.2, while caregivers reported a mean of neuropsychiatric symptoms of 18.2. A total of 73% (*n* = 46) of the dyads used assistive technology. The mean age of caregivers was 62.5 years, 68% (*n* = 43) were women and 35% (*n* = 22) were married to the PwD, while 62% (*n* = 39) were adult children of the PwD. Caregivers reported a mean QoL-AD of 40.2, EQ-5-D VAS scale for health of 29.9, relative stress scale of 15.4, and geriatric depression scale of 5.3. We found no significant difference between the demographic and clinical variables of the dyads when comparing the group accepting Alight (*n* = 8) with the group not accepting Alight (*n* = 55) (Table 1).

**Table 1:** Characteristics of the sample offered Alight application in the LIVE@Home.Path trial.

	**Total sample (*n* = 63)**	**Accepting Alight (*n* = 8)**	**Not accepting Alight (*n* = 55)**	*p*-value^*^
**Person with dementia**
Age, mean (S.D.)	82.2 (6.5)	79.2 (5.8)	82.7 (6.6)	0.16
Female gender, *n* (%)	39 (61.9)	5 (62.5)	34 (61.8)	0.97
Living alone, *n* (%)	35 (55.6)	3 (37.5)	32 (58.2)	0.27
Type dementia				0.56
Alzheimer's dementia, *n* (%)	27 (42.8)	3(37.5)	24 (43.6)	
Vascular dementia, *n* (%)	2 (3.17)	0 (0%)	2 (3.6)	
Lewy body dementia, *n* (%)	1 (1.59)	0 (0)	1 (1.8)	
Parkinson's dementia, *n* (%)	2 (3.17)	1 (12.5)	1 (1.8)	
Mixed dementia, *n* (%)	31 (49.2)	4 (50%)	27 (49.1)	
MMSE, range 0–30, mean (S.D.)	19.6 (4.3)	17.3 (4.7)	20.0 (4.2)	0.13
Missing, *n* (%)	7(9.5)	1 (12.5)	6 (10.9)	
FAST Range 1–7, *n* (%)				0.99
Mild (3–4)	39 (70.9)	5 (62.5)	34 (61.8)	
Moderate (5)	8 (14.5)	1 (12.5)	7 (12.7)	
Severe (6–7)	8 (14.5)	1 (12.5)	7 (12.7)	
Missing, *n* (%)	8 (14.5)	1 (12.5)	7 (12.7)	
**ADL**
P-ADL (range 6–30), mean (S.D.)	11.0 (3.5)	12.5 (5.0)	10.7 (3.5)	0.22
Missing, *n* (%)	2	0	2	
I-ADL (range 8–31), mean (S.D.)	20.7 (6.0)	21.8 (6.3)	20.6 (6.0)	0.63
Missing, *n* (%)	3	0	3	
Use of assistive technology, *n* (%)	46 (73.0)	4 (50)	42 (76.4)	0.12
NPI total score, range 0–144, mean (S.D.)	18.2 (17.2)	22.4(13.0)	17.6(17.7)	0.47
Missing, *n* (%)	2	0	2	
QoL-AD patient reported, range 13–52, mean (S.D.)	37.2 (5.4)	35.3 (5.8)	37.5 (5.4)	0.27
Missing	3 (4.7)	0 (0.0)	3 (4.7)	
EQ-5-D VAS scale health, range 0–100, mean (S.D.)	76.2 (18.0)	70.0 (20.5)	76.2 (18.0)	0.37
Missing, *n* (%)	2 (3.1)	0 (0.0)	2 (3.6)	
**Caregiver**
Age, mean (S.D.)	62.5(12.5)	64.5 (10.2)	62.3 (12.9)	0.64
Female gender, *n* (%)	43 (68.3)	6 (75%)	37 (67.3)	0.66
CG kinship				0.78
Spouse, *n* (%)	22 (34.9)	4 (50%)	18 (32.7)	
Child, *n* (%)	39 (61.9)	4 (50%)	35 (63.6)	
Other (friend, formal advocate), *n* (%)	2 (3.2)	0 (0%)	2 (3.6)	
QoL-AD, range 13–52, mean (S.D.)	40.4 (6.1)	39.9 (8.5)	40.5 (5.7)	0.79
Missing, *n* (%)	2 (3.2)	0 (0)	2 (3.6)	
EQ-5-D VAS scale health, range 0–100, mean (S.D.)	29.9 (38.1)	32.9 (45.7)	29.5 (37.4)	0.81
RSS, range 0–60, mean (S.D.)	15.4 (9.8)	20.4 (5.6)	14.7 (10.0)	0.15
GDS, range 0–30, mean (S.D.)	5.3 (4.9)	6.5 (5.6)	5.1 (4.8)	0.46
	1	0	1	

MMS, Mini Mental State Examination; FAST, Functional Assessment Staging Test; ADL, Activity of Daily Living; P-ADL, Personal Activities of Daily Living; I-ADL, Instrumental Activities of Daily Living; NPI, Neuropsychiatric Inventory; QoL-AD, Quality of Life in Alzheimer's disease scale; EQ-5-D VAS, European Quality of Life 5 dimensions visual analog scale; RSS, Relative Stress Scale; GDS, Geriatric Depression Scale; S.D., Standard deviation.

Missing equals zero if not otherwise specified.

* *p*-value for difference, t-test for comparison of continuous variables, chi-square for categorical variables.

**Figure 4:**
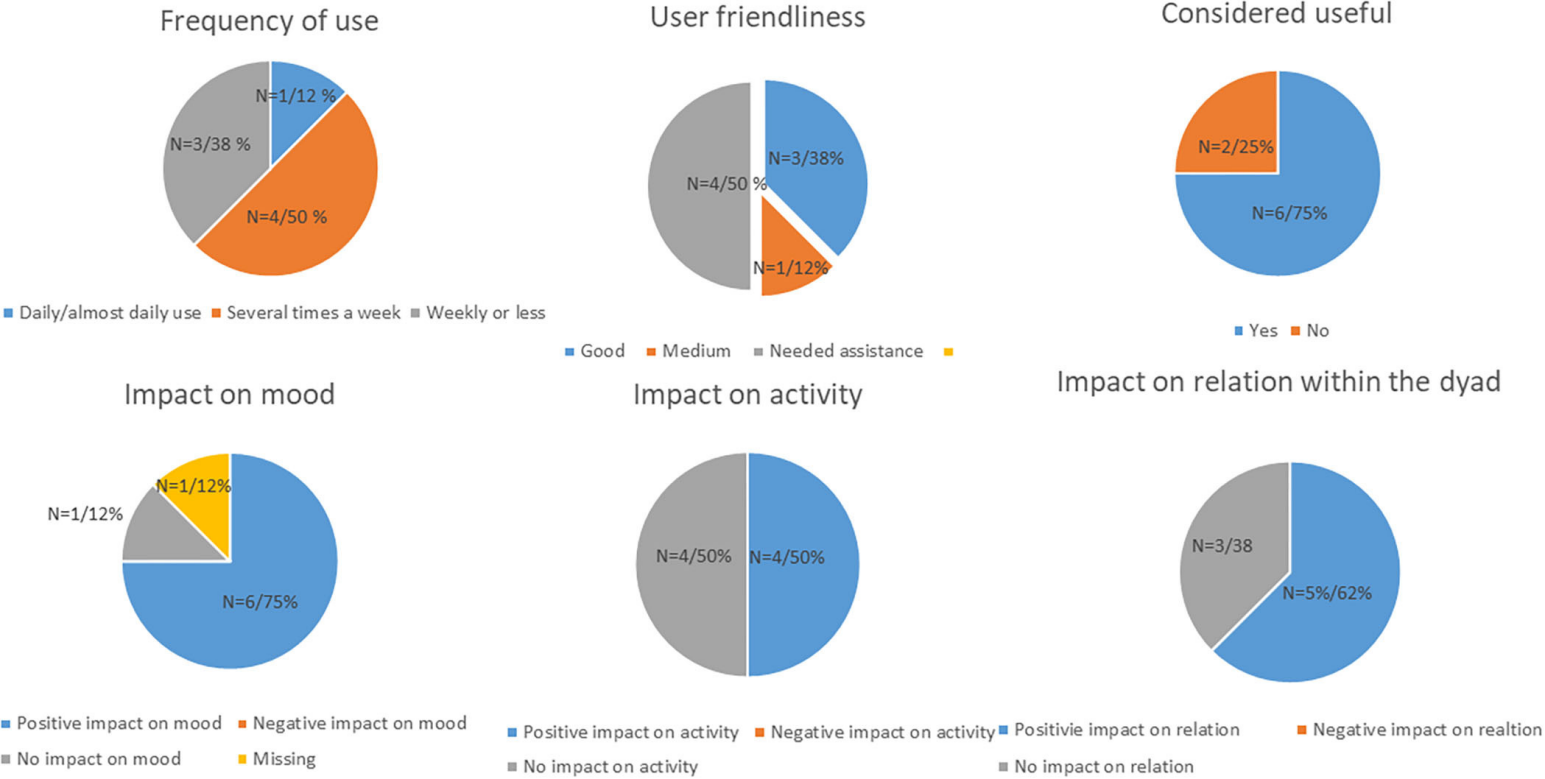
Data on adoption and feasibility of the Alight application tested in 8 dyads of PwD and caregivers in the LIVE@Home.Path trial.

Six of the eight dyads used the application on the hospital's iPad, the remaining two dyads used their personal iPad. Dyads using Alight reported overall high adoption of the application, as 50% (*n* = 4) used it several times a week, and 12% (*n* = 1) reported daily use (Figure 4). Stratifying adoption by cohabitating status revealed that 33% (one of three) of the dyads with PwD living alone reported use several times a week, while the corresponding number among dyads with co-residing PwD and caregiver was 82% (four of five) (data not shown). The feasibility was also high, 50% (*n* = 4) reported good or medium user friendliness, while the latter 50% (*n* = 4) needed assistance in use by their informal caregiver. All participants reported that the content was personally tailored to their needs, and 75% (*n* = 6) found it useful to engage with the application. A total of 75% (*n* = 6) of the dyads found that the application had a positive impact on mood, 50% (*n* = 4) found that it had a positive impact on activity, while 62% (*n* = 5) reported a positive impact on communication and relation within the dyad. On a scale of 1–10 (*n* = 8), overall mean satisfaction was 8.0 (95% CI: 5.5, 10.5), while the likelihood of recommending the application to others was 9.1 (95% CI: 8.2, 10.1) (data not shown in Table 1).

We categorized the comments from the open questions in the self-developed feedback form into two main groups: feedback on (1) content and (2) obstacles for use. Several dyads reported that the application gave them a momentary sense of mastery and self-efficacy as they experienced that they could acquire new technological skills. One dyad found that it aided in recollecting good memories, thereby shifting the focus away from the impaired health. One dyad particularly enjoyed using the application as a tool for reminiscence together with their common friends, while it inspired another dyad to take up dancing. On the other hand, several dyads reported that despite simple procedures, the PwD did not manage to use the application independently due to digital illiteracy, and one PwD was hindered in using the application alone due to impaired vision. These obstacles limited the frequency of use for some of the dyads. This impression was also confirmed by the music therapist visiting the dyads before the application was installed, she found that some PwDs were unfamiliar with applying the right “touch” on the iPads to manage the screen. Furthermore, she found it challenging to update the application in homes without domestic WiFi, and had to bring it to an area with WiFi to install required updates.

## Discussion

This study describes the participatory design process of a prototype music application for patients affiliated with a gerontopsychiatric hospital and the evaluation of acceptability, adoption, and feasibility when used as a digital musical memory book for dyads of home-dwelling PwD and caregivers in a clinical trial in municipal dementia care. It contributes methodologically to the field of participatory design and mHealth interventions by demonstrating a specific approach that succeeded in involving researchers, industry partners, health care practitioners, and most importantly, PwD and caregivers in the design decisions. When the application was offered as part of a multicomponent intervention, we found that 13% of the dyads accepted use, and these dyads did not differ on demographic and clinical characteristics compared to those not accepting. Overall, the adoption and feasibility of the application were high, as 63% reported daily use or use several times a week, high satisfaction, and likelihood of recommending the application to others. These results are of key importance to stakeholders and health care providers in dementia care, as they suggest a high potential for reach and utilization of mobile music interventions to PwD and caregivers residing at home, as well as providing a cost-effective and personalized tool for professional engagement of PwD and caregivers, regardless of care level.

While eHealth is a broad term describing how ICT can support health (8), telehealth more specifically concerns the use of technological health services over a distance, and telemedicine describes the use of remote clinical services directed explicitly at patients. Similarly, telecare refers to a certain method to monitor fragile patients by alarms, sensors, and other assistive technology (35). All these remote services involve interaction with health care personnel, and this contact can for example be provided *via* applications for smartphones and tablets. This can be in contrast to the concept of mHealth, in which the users are not necessarily considered as “patients”, but rather “consumers” of mobile self-care *via* applications, enabling them to monitor and enhance their own health (36). Evidently, applications for PwD can be used with or without the involvement of health care personnel, of which the exploration of Alight in the LIVE@Home.Path is an example of the first. In our study, a music therapist with a university master's degree assessed and evaluated the PwDs medical condition and musical preferences, before she created a therapy session to be designed in the application based on her expert knowledge of the therapeutic factors of music in dementia (23). In contrast to the impaired short-term memory characteristic of dementia, musical memory is often well preserved even in the late stages, which can be utilized to maintain a sense of identity for the ones affected throughout the dementia course (5). This knowledge is of utmost importance when providing clinical services to people with dementia because the syndrome is associated with loss of abilities and roles, thereby increasing the dependency on others. Using Alight to deliver personalized remote music interventions to patients at home could thus serve as an example of telemedicine, by bringing the therapy to the patient outside the health care system. Applications as an adjunctive element of care are increasingly used in the gerontopsychiatric field (37) and exemplified by a recent study on suicidal behavior in older adults in which a multicomponent intervention included an application that reinforced cognitive strategies for emotion regulation developed with a therapist in inpatient sessions (38).

Our group has previously shown that 75% of PwDs in the baseline sample of the LIVE@Home.Path trial used assistive technology and/or telecare, defined as any device or system that maintains or improves the person's ability to perform tasks they would otherwise be unable to or increase the ease or safety of tasks performed (39). Nevertheless, the majority had traditional equipment such as stove guards, social alarms, and calendar support, while only one dyad utilized a mHealth application for tracking (39). In the Danish “Rehabilitation in Alzheimer's disease using Cognitive support Technology” (ReACT) project, researchers and users co-designed an application promoting self-management for PwDs by enhancing memory and structuring of daily activities (40, 41). Comparable to our findings of 13% accepting Alight in the present study, their explorative study of adoption and use patterns of the ReACT application revealed that a total of 16% of 112 PwD recruited from memory clinics adopted use, defined as a minimum period of 90 days between first and last use evaluated by data logs. A shorter time from diagnosis and caregiver activating the application increased the likelihood of adoption (42). Several reasons can explain the relatively low acceptance rate of Alight in the LIVE@Home.Path trial. In ReACT project, the PwDs were solely offered the application under study, while in our trial, the application was offered as part of a multicomponent intervention. Consequently, the interest in Alight might be challenged by other tailored, yet time-consuming, services, spanning from other assistive technologies to educational courses and visiting friends. Moreover, In ReACT project, a dyad of PwD and caregivers were included in 88% of the cases, and most of these caregivers were spouses. We found that the acceptance rate varied substantially by cohabiting status. Among dyads in which the PwD lived alone, the acceptance rate was 8.6% while the corresponding number among dyads with co-residing PwD was 17.9%. We therefore suggest that the fairly low acceptance rate in our study is also partly explained by half of the PwD residing alone in our sample. Furthermore, the researchers of the ReACT project discuss the well-known challenges of non-adoption and attrition of digital health interventions in general, and highlight the importance of contextual factors, timely introduction, and support of caregivers when implementing assistive technology in dementia care (42). This is in line with other findings from the LIVE@Home.Path, in which only a minority of the caregivers reported increased interest in new devices during the COVID-19 restrictions, suggesting that they might consider assistive technology as an obstacle rather than a tool for independence in adapting to the new and changing pandemic context (34).

Similarly, the timely introduction is crucial for the adoption and feasibility of new technology for persons with progressive neurodegenerative disorders, such as dementia. Even though Alight was developed through a rigorous and iterative participatory design process involving health care personnel, patients, and caregivers in prototyping and testing, as much as 50% of the accepting dyads had difficulties managing it independently. Besides impaired vision, this was mainly caused by unfamiliarity with navigating the iPad and applying the right “touch” on the screen. Digital immigrants denote persons who grew up before the digital age, and therefore must acquire these skills in a “new digital world”. Impaired ability to learn is a hallmark of dementia, and in general, technology is often introduced too late in the dementia course to support the loss of independence (43). Optimistically, this might change over the next decades when older adults are likely more familiar with the use of applications and touch screens before disease onset. This is underscored by recent findings from a postal survey among adults over 60 years in Germany, showing that the youngest ones had higher technical readiness and used health apps more frequently compared to the oldest old (44). Meanwhile, we suggest that the use of the Alight application in the LIVE@Home.Path served as a magnifying glass in the PwDs hands, the participants who could already use or were able to acquire new technological skills experienced positive feelings of mastery and self-efficacy, while for those not ready, it served as yet another confirmation of loss of function and dependency.

The major strength of the study is the description of the 4-year participatory design process of a prototype application with end-user involvement at every stage of the iterative process, including two rounds of prototyping and testing in a real-world setting. Throughout the study, we worked together as a multidisciplinary team, including experts from industry, computer engineering, researcher, health care, and user background. An additional strength is the evaluation of acceptability, adoption, and feasibility in a large, stepped wedge multicomponent randomized controlled trial with dyads of PwD and caregivers in municipal dementia care. Nonetheless, we used convenience sampling to recruit the dyads from the health care services, which may limit the generalizability of our findings to PwD supported by formal and informal caregivers. The COVID-19 pandemic and restrictions led to a smaller sample being offered Alight than scheduled, which possibly hindered us from determining distinct clinical and demographical characteristics of dyads accepting use. Furthermore, the nature of the multicomponent LIVE intervention unlabeled us to disentangle and evaluate a possible effect of the music intervention provided with Alight on relevant clinical outcomes. Due to the restricted dimension of the material and the amount of information obtained, we did not deem it feasible to perform formal qualitative analysis, such as thematic or narrative analysis, on the answers to the open-ended questions. A final limitation is that we did not utilize any recognized evaluation tool or guidelines for reporting of mHealth interventions.

In conclusion, the adoption and feasibility of the prototype music application Alight were high among home-dwelling persons with dementia, and the accepting dyads did not differ on demographic and clinical variables compared to those not reached. This suggests a high potential for utilization in dementia care. Future studies should evaluate the impact of mobile music interventions adjunctive to other elements of care for PwD and caregivers on relevant clinical outcomes, such as cognition, neuropsychiatric symptoms, quality of life, caregiver burden and relation, as well as resource utilization. A participatory design process is valuable for safeguarding acceptability, adoption, and feasibility of future mHealth applications in dementia care, and accurate labeling of the application can permit the users to select an application that fits their current and future needs and resources.

## Data availability statement

The raw data supporting the conclusions of this article will be made available to other researchers by the corresponding author upon reasonable request.

## Ethics statement

The studies involving human participants were reviewed and approved by Regional Committee for Medical and Health Research Ethics, North Norway (2019/385). The patients/participants provided their written informed consent to participate in this study.

## Author contributions

LB, MG, and BH conducted the LIVE@Home.Path trial, developed the intervention including the second prototyping and testing of the Alight application, and evaluated the acceptability and feasibility of Alight in the trial. LB and MG conducted descriptive analyses. SK, KH, and KM initiated the development of the Alight application, held workshops, and conducted the first prototyping and testing. JT provided input on and drafted the app development process. LB drafted the manuscript, while all other authors revised it critically for important intellectual content. All authors contributed to the conception and design of the final study, as well as the analyses and interpretation of the data. All authors contributed to the article and approved the submitted version.

## Funding

This project received funding from the Helse Vest Regional Health Trust (100,000 NOK), the Ekstra Stiftelsen/DAM (260,000 NOK), and the InnoMed (200,000 NOK), covering the payroll compensation for Soundio and VIS, and the cost of workshops and iPads. NKS Olaviken Gerontopsychiatric Hospital covered payroll compensation for staff participating in workshops and interviews. Research Council of Norway funded the LIVE@Home.Path trial including LB and MGs positions, sponsor code 273581.

## Conflict of interest

The authors declare that the research was conducted in the absence of any commercial or financial relationships that could be construed as a potential conflict of interest.

## Publisher's note

All claims expressed in this article are solely those of the authors and do not necessarily represent those of their affiliated organizations, or those of the publisher, the editors and the reviewers. Any product that may be evaluated in this article, or claim that may be made by its manufacturer, is not guaranteed or endorsed by the publisher.
