# Supplementary material for: The acceptability, adoption, and feasibility of a music application developed using participatory design for home-dwelling persons with dementia and their caregivers. The “Alight” app in the LIVE@Home.Path trial
Source: Front Psychiatry. 2022 Aug 18;13:949393. doi: 10.3389/fpsyt.2022.949393 (PMC9433972; doi:10.3389/fpsyt.2022.949393)
Supplement: Supplementary file 1 [file Data_Sheet_1.docx]

**Interview guide after prototyping 1 and testing 1 (2018):**

Questions to the patient or his formal or informal caregiver:

1. How did you use the Alight application?
2. How did you experience the use of Alight? Was it positive? Negative?
3. Did you experience any change over time un use? More/less independent use? Others?
4. What is our opinion on the design of the Alight application and the technical solution? Do you have any suggestion on how to improve it?
5. Did you find it useful to engage in the Alight application?
