# Supplementary material for: The acceptability, adoption, and feasibility of a music application developed using participatory design for home-dwelling persons with dementia and their caregivers. The “Alight” app in the LIVE@Home.Path trial
Source: Front Psychiatry. 2022 Aug 18;13:949393. doi: 10.3389/fpsyt.2022.949393 (PMC9433972; doi:10.3389/fpsyt.2022.949393)
Supplement: Supplementary file 2 [file Data_Sheet_2.docx]

**LIVE@Home.Path Trial identification number:…………..**

**Have you used the Alight application?**

__ Yes, daily or close to daily

__ Yes, several times a week

__ Yes, weekly or more seldom

__ No, I have not used Alight, because (give a reason for not using the application):

…………………………………………………………………………………………………

…………………………….

**How do you rate the user friendliness of the Alight application:**

__ Good (easy) __ Icons were appropriately sized

__ Medium (a bit challenging) __ Icons were to small

__ Poor (unusable)

__ I needed assistance when using the application

Do you have other comments regarding the user friendliness of the Alight application?

…………………………………………………………………………………………………

………………………………………

**Was the content of the Alight application tailored to your needs?**

__ To a maximum extent

__ To a large extent

__ To some extent

__ Not at all

Do you have other comments regarding the content of the Alight application?

…………………………………………………………………………………………………

……………………………………………

**Do you consider the Alight application useful in your situation?**

__ No, it was not useful because………………………………………………………………………………………

__ Yes, it was useful because………………………………………………………………………………………

**Do you consider that the Alight application had impact on your mood?**

_ No

_Yes, positive impact on mood

_ Yes, negative impact on mood

**Do you consider that the Alight application had impact on your level of activity?**

_No

_Yes, positive impact on activity

_Yes, negative impact on activity

**Do you consider that the Alight application had impact on communication and relation with your relative?**

_No

_ Yes, positive impact on communication and relation

_Yes, negative impact on communication and relation

**On a scale from 1-10, how satisfied are you with using the Alight application?**

Not satisfied at all Maximum satisfied

1---------2---------3---------4---------5---------6---------7---------8---------9---------10

**On a scale 1-10, how likely is it that you will recommend the Alight application to someone in your situation?**

Not likely at all Will definitely recommend it

1---------2---------3---------4---------5---------6---------7---------8---------9---------10

**We highly appreciate all other feedback regarding the Alight application:**

**……………………………………………………………………………………………………………………………………………………………………………………………………………………………………………………………………………………………………………………………………………………………………………………………………………………………………………………………………………………………………………………………………………………………………………………………………………………………………………………………………………………………………………………………………………………………………………………………………………………………………………………………………………….**

**Thank you for your time and participation in the LIVE@Home.Path trial**
